# (*E*)-3-(2-Chloro­phen­yl)-2-[(2-formylphen­oxy)meth­yl]prop-2-ene­nitrile

**DOI:** 10.1107/S1600536812008410

**Published:** 2012-03-03

**Authors:** N. Manikandan, S. Murugavel, D. Kannan, M. Bakthadoss

**Affiliations:** a Department of Physics, Bharathidasan Engineering College, Nattrampalli, Vellore 635 854, India; b Department of Physics, Thanthai Periyar Government Institute of Technology, Vellore 632 002, India; c Department of Organic Chemistry, University of Madras, Maraimalai Campus, Chennai 600 025, India

## Abstract

In the title compound, C_17_H_12_ClNO_2_, the dihedral angle between the two benzene rings is 42.9 (1)°. There are no sgnificant inter­molecular inter­actions.

## Related literature

For background to the synthetic procedure, see: Bakthadoss & Murugan (2010 ▶). For related structures, see: Manikandan *et al.* (2012 ▶); Prasanna *et al.* (2011 ▶). 
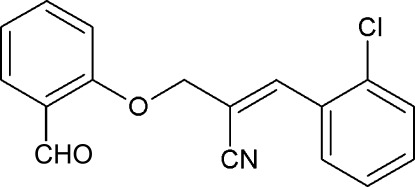


## Experimental

### 

#### Crystal data

C_17_H_12_ClNO_2_
*M* *_r_* = 297.73
Triclinic, 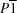

*a* = 7.5022 (4) Å
*b* = 7.8301 (4) Å
*c* = 13.2379 (8) Å
α = 75.470 (3)°
β = 84.696 (2)°
γ = 70.935 (2)°
*V* = 711.43 (7) Å^3^
*Z* = 2
Mo *K*α radiation
μ = 0.27 mm^−1^
*T* = 293 K
0.24 × 0.21 × 0.15 mm

#### Data collection

Bruker APEXII CCD diffractometer
Absorption correction: multi-scan (*SADABS*; Sheldrick, 1996 ▶) *T*_min_ = 0.937, *T*_max_ = 0.960
14438 measured reflections
3433 independent reflections
2685 reflections with *I* > 2σ(*I*)
*R*_int_ = 0.029

#### Refinement

*R*[*F* ^2^ > 2σ(*F* ^2^)] = 0.041
*wR*(*F* ^2^) = 0.114
*S* = 1.04
3433 reflections
190 parameters
H-atom parameters constrained
Δρ_max_ = 0.20 e Å^−3^
Δρ_min_ = −0.19 e Å^−3^

### 

Data collection: *APEX2* (Bruker, 2004 ▶); cell refinement: *APEX2* and *SAINT* (Bruker, 2004 ▶); data reduction: *SAINT* and *XPREP* (Bruker, 2004 ▶); program(s) used to solve structure: *SHELXS97* (Sheldrick, 2008 ▶); program(s) used to refine structure: *SHELXL97* (Sheldrick, 2008 ▶); molecular graphics: *ORTEP-3* (Farrugia, 1997 ▶); software used to prepare material for publication: *SHELXL97* and *PLATON* (Spek, 2009 ▶).

## Supplementary Material

Crystal structure: contains datablock(s) global, I. DOI: 10.1107/S1600536812008410/bt5830sup1.cif

Structure factors: contains datablock(s) I. DOI: 10.1107/S1600536812008410/bt5830Isup2.hkl

Supplementary material file. DOI: 10.1107/S1600536812008410/bt5830Isup3.cml

Additional supplementary materials:  crystallographic information; 3D view; checkCIF report

## Figures and Tables

**Fig. 1.**
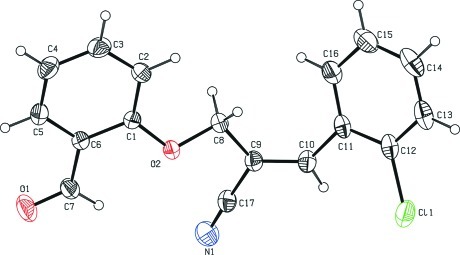
Molecular structure of the title compound showing displacement ellipsoids at the 30% probability level. H atoms are presented as a small spheres of arbitrary radii.

## supplementary crystallographic information

### Comment

The title compound is a stereodefined trisubstituted olefin, synthesized from the corresponding bromoderivative of Baylis–Hillman adduct with salicylaldehyde *via* simple SN2 reaction in good yields. This *o*-salicyladehyde derivative is an important precursor for many heterocyclic frameworks (Bakthadoss & Murugan, 2010).

The title compound comprises a benzaldehyde moiety connected to a chlorophenyl ring through a chain formed by a methoxy methyl and a propenenitrile group. The X-ray analysis confirms the molecular structure and atom connectivity as illustrated in Fig. 1.

The dihedral angle between the two aromatic rings is 42.9 (1)°. The propenenitrile (N1/C17/C8–C11) plane forms dihedral angles of 12.4 (1)° and 36.0 (1)°, respectively, with the formyl phenyl and chloro phenyl rings. The Cl1 atom deviates from the plane of attached ring by 0.019 (1) Å. The bond length C9—C17 [1.443 (2) Å] is significantly shorter than the expected value for a C—C single bond because of conjugation effects (Prasanna *et al.*, 2011). The carbonitrile side chain (C9—C17—N1) is almost linear, with the angle around the central C atom being 178.1 (2)°. The geometric parameters of the title molecule agree well with those reported for similar structures (Manikandan *et al.*, 2012; Prasanna *et al.*, 2011).

### Experimental

A solution of salicylaldehyde (1.0 mmol, 0.122 g) and potassium carbonate (1.5 mmol, 0.207 g) in acetonitrile solvent was stirred for 15 min at room temperature. To this solution, (*E*)-2-(bromomethyl)-3-(2-chlorophenyl)prop-2-enenitrile (1.2 mmol, 0.308 g) was added drop wise till the addition is complete. After the completion of the reaction, as indicated by TLC, acetonitrile was evaporated. EtOAc (15 ml) and water (15 ml) were added to the crude mass. The organic layer was dried over anhydrous sodium sulfate. Removal of solvent led to a crude product, which was purified through a pad of silica gel (100–200 mesh) using ethyl acetate and hexanes (1:9) as solvents. The pure title compound was obtained as a colorless solid (0.270 g, 90% yield). Recrystallization was carried out using ethyl acetate as solvent.

### Refinement

H atoms were positioned geometrically, with C—H = 0.93–0.97 Å and constrained to ride on their parent atom, with *U*_iso_(H) = 1.2*U*_eq_(C).

### Crystal data

**Table:**

C_17_H_12_ClNO_2_	*Z* = 2
*M**_r_* = 297.73	*F*(000) = 308
Triclinic, *P*1	*D*_x_ = 1.390 Mg m^−^^3^
Hall symbol: -P 1	Mo *K*α radiation, λ = 0.71073 Å
*a* = 7.5022 (4) Å	Cell parameters from 3463 reflections
*b* = 7.8301 (4) Å	θ = 2.8–28.1°
*c* = 13.2379 (8) Å	µ = 0.27 mm^−^^1^
α = 75.470 (3)°	*T* = 293 K
β = 84.696 (2)°	Block, colourless
γ = 70.935 (2)°	0.24 × 0.21 × 0.15 mm
*V* = 711.43 (7) Å^3^	

### Data collection

**Table:**

Bruker APEXII CCD diffractometer	3433 independent reflections
Radiation source: fine-focus sealed tube	2685 reflections with *I* > 2σ(*I*)
Graphite monochromator	*R*_int_ = 0.029
Detector resolution: 10.0 pixels mm^-1^	θ_max_ = 28.1°, θ_min_ = 2.8°
ω scans	*h* = −9→9
Absorption correction: multi-scan (*SADABS*; Sheldrick, 1996)	*k* = −10→10
*T*_min_ = 0.937, *T*_max_ = 0.960	*l* = −17→17
14438 measured reflections	

### Refinement

**Table:**

Refinement on *F*^2^	Primary atom site location: structure-invariant direct methods
Least-squares matrix: full	Secondary atom site location: difference Fourier map
*R*[*F*^2^ > 2σ(*F*^2^)] = 0.041	Hydrogen site location: inferred from neighbouring sites
*wR*(*F*^2^) = 0.114	H-atom parameters constrained
*S* = 1.04	*w* = 1/[σ^2^(*F*_o_^2^) + (0.053*P*)^2^ + 0.159*P*] where *P* = (*F*_o_^2^ + 2*F*_c_^2^)/3
3433 reflections	(Δ/σ)_max_ < 0.001
190 parameters	Δρ_max_ = 0.20 e Å^−^^3^
0 restraints	Δρ_min_ = −0.19 e Å^−^^3^

### Special details

**Table:**

Geometry. All e.s.d.'s (except the e.s.d. in the dihedral angle between two l.s. planes) are estimated using the full covariance matrix. The cell e.s.d.'s are taken into account individually in the estimation of e.s.d.'s in distances, angles and torsion angles; correlations between e.s.d.'s in cell parameters are only used when they are defined by crystal symmetry. An approximate (isotropic) treatment of cell e.s.d.'s is used for estimating e.s.d.'s involving l.s. planes.
Refinement. Refinement of *F*^2^ against ALL reflections. The weighted *R*-factor *wR* and goodness of fit *S* are based on *F*^2^, conventional *R*-factors *R* are based on *F*, with *F* set to zero for negative *F*^2^. The threshold expression of *F*^2^ > σ(*F*^2^) is used only for calculating *R*-factors(gt) *etc*. and is not relevant to the choice of reflections for refinement. *R*-factors based on *F*^2^ are statistically about twice as large as those based on *F*, and *R*-factors based on ALL data will be even larger.

### Fractional atomic coordinates and isotropic or equivalent isotropic displacement parameters (Å^2^)

**Table:**

	*x*	*y*	*z*	*U*_iso_*/*U*_eq_	
Cl1	0.31341 (8)	0.23140 (7)	0.01456 (3)	0.07011 (17)	
N1	0.1141 (2)	−0.1357 (2)	0.40658 (12)	0.0660 (4)	
O1	0.3876 (2)	−0.21291 (18)	0.75288 (10)	0.0765 (4)	
O2	0.28615 (15)	0.15782 (13)	0.48411 (7)	0.0436 (2)	
C1	0.32059 (19)	0.22280 (18)	0.56458 (10)	0.0364 (3)	
C2	0.3173 (2)	0.4030 (2)	0.55536 (12)	0.0471 (4)	
H2	0.2830	0.4913	0.4928	0.057*	
C3	0.3655 (3)	0.4509 (2)	0.64002 (14)	0.0562 (4)	
H3	0.3639	0.5724	0.6336	0.067*	
C4	0.4160 (3)	0.3238 (2)	0.73390 (13)	0.0559 (4)	
H4	0.4513	0.3578	0.7896	0.067*	
C5	0.4132 (2)	0.1464 (2)	0.74357 (11)	0.0474 (4)	
H5	0.4442	0.0603	0.8071	0.057*	
C6	0.36463 (19)	0.09286 (18)	0.66019 (10)	0.0377 (3)	
C7	0.3555 (2)	−0.0949 (2)	0.67304 (12)	0.0479 (4)	
H7	0.3225	−0.1251	0.6153	0.057*	
C8	0.1837 (2)	0.2928 (2)	0.39872 (11)	0.0428 (3)	
H8A	0.2576	0.3715	0.3627	0.051*	
H8B	0.0670	0.3705	0.4233	0.051*	
C9	0.1425 (2)	0.1907 (2)	0.32654 (11)	0.0407 (3)	
C10	0.1257 (2)	0.2525 (2)	0.22304 (11)	0.0453 (3)	
H10	0.0988	0.1754	0.1876	0.054*	
C11	0.1448 (2)	0.4273 (2)	0.15926 (11)	0.0450 (3)	
C12	0.2291 (2)	0.4332 (2)	0.05996 (12)	0.0505 (4)	
C13	0.2510 (3)	0.5949 (3)	−0.00219 (14)	0.0639 (5)	
H13	0.3080	0.5953	−0.0676	0.077*	
C14	0.1887 (3)	0.7543 (3)	0.03273 (17)	0.0725 (6)	
H14	0.2040	0.8635	−0.0090	0.087*	
C15	0.1029 (3)	0.7550 (3)	0.12961 (16)	0.0693 (5)	
H15	0.0605	0.8643	0.1528	0.083*	
C16	0.0804 (3)	0.5936 (2)	0.19183 (13)	0.0562 (4)	
H16	0.0212	0.5955	0.2566	0.067*	
C17	0.1252 (2)	0.0091 (2)	0.37263 (11)	0.0463 (3)	

### Atomic displacement parameters (Å^2^)

**Table:**

	*U*^11^	*U*^22^	*U*^33^	*U*^12^	*U*^13^	*U*^23^
Cl1	0.0911 (4)	0.0811 (3)	0.0395 (2)	−0.0294 (3)	0.0038 (2)	−0.0151 (2)
N1	0.0812 (11)	0.0554 (9)	0.0620 (9)	−0.0321 (8)	0.0090 (8)	−0.0041 (7)
O1	0.1249 (12)	0.0518 (7)	0.0519 (7)	−0.0394 (8)	−0.0187 (7)	0.0113 (6)
O2	0.0599 (6)	0.0349 (5)	0.0322 (5)	−0.0102 (4)	−0.0091 (4)	−0.0044 (4)
C1	0.0398 (7)	0.0358 (7)	0.0329 (6)	−0.0113 (5)	0.0013 (5)	−0.0083 (5)
C2	0.0612 (9)	0.0358 (7)	0.0424 (8)	−0.0170 (7)	0.0022 (7)	−0.0043 (6)
C3	0.0757 (11)	0.0435 (8)	0.0598 (10)	−0.0286 (8)	0.0056 (8)	−0.0197 (7)
C4	0.0689 (11)	0.0625 (10)	0.0472 (9)	−0.0272 (8)	0.0005 (7)	−0.0242 (8)
C5	0.0562 (9)	0.0514 (9)	0.0344 (7)	−0.0170 (7)	−0.0019 (6)	−0.0089 (6)
C6	0.0424 (7)	0.0352 (7)	0.0341 (7)	−0.0121 (6)	0.0002 (5)	−0.0062 (5)
C7	0.0610 (9)	0.0390 (8)	0.0421 (8)	−0.0176 (7)	−0.0056 (7)	−0.0025 (6)
C8	0.0504 (8)	0.0388 (7)	0.0339 (7)	−0.0099 (6)	−0.0036 (6)	−0.0037 (5)
C9	0.0417 (7)	0.0429 (7)	0.0354 (7)	−0.0138 (6)	0.0005 (5)	−0.0050 (6)
C10	0.0520 (8)	0.0507 (8)	0.0358 (7)	−0.0215 (7)	−0.0031 (6)	−0.0069 (6)
C11	0.0469 (8)	0.0512 (9)	0.0339 (7)	−0.0172 (7)	−0.0113 (6)	0.0017 (6)
C12	0.0520 (9)	0.0601 (10)	0.0358 (7)	−0.0200 (7)	−0.0105 (6)	0.0020 (7)
C13	0.0614 (10)	0.0752 (13)	0.0447 (9)	−0.0263 (9)	−0.0070 (8)	0.0132 (8)
C14	0.0733 (13)	0.0589 (12)	0.0736 (13)	−0.0287 (10)	−0.0198 (10)	0.0222 (10)
C15	0.0788 (13)	0.0479 (10)	0.0730 (13)	−0.0153 (9)	−0.0219 (10)	0.0017 (9)
C16	0.0621 (10)	0.0509 (9)	0.0478 (9)	−0.0124 (8)	−0.0107 (7)	−0.0013 (7)
C17	0.0510 (8)	0.0507 (9)	0.0366 (7)	−0.0191 (7)	0.0028 (6)	−0.0062 (6)

### Geometric parameters (Å, º)

**Table:**

Cl1—C12	1.7353 (18)	C8—C9	1.499 (2)
N1—C17	1.137 (2)	C8—H8A	0.9700
O1—C7	1.2004 (18)	C8—H8B	0.9700
O2—C1	1.3672 (16)	C9—C10	1.3367 (19)
O2—C8	1.4199 (16)	C9—C17	1.443 (2)
C1—C2	1.378 (2)	C10—C11	1.460 (2)
C1—C6	1.3992 (18)	C10—H10	0.9300
C2—C3	1.379 (2)	C11—C16	1.395 (2)
C2—H2	0.9300	C11—C12	1.402 (2)
C3—C4	1.378 (2)	C12—C13	1.376 (2)
C3—H3	0.9300	C13—C14	1.364 (3)
C4—C5	1.369 (2)	C13—H13	0.9300
C4—H4	0.9300	C14—C15	1.381 (3)
C5—C6	1.390 (2)	C14—H14	0.9300
C5—H5	0.9300	C15—C16	1.377 (3)
C6—C7	1.460 (2)	C15—H15	0.9300
C7—H7	0.9300	C16—H16	0.9300
			
C1—O2—C8	116.56 (10)	H8A—C8—H8B	108.5
O2—C1—C2	123.98 (12)	C10—C9—C17	117.74 (14)
O2—C1—C6	115.96 (11)	C10—C9—C8	125.17 (13)
C2—C1—C6	120.05 (13)	C17—C9—C8	117.06 (12)
C1—C2—C3	119.14 (14)	C9—C10—C11	127.43 (14)
C1—C2—H2	120.4	C9—C10—H10	116.3
C3—C2—H2	120.4	C11—C10—H10	116.3
C4—C3—C2	121.89 (14)	C16—C11—C12	117.11 (14)
C4—C3—H3	119.1	C16—C11—C10	122.99 (14)
C2—C3—H3	119.1	C12—C11—C10	119.90 (14)
C5—C4—C3	118.66 (15)	C13—C12—C11	121.65 (17)
C5—C4—H4	120.7	C13—C12—Cl1	118.70 (14)
C3—C4—H4	120.7	C11—C12—Cl1	119.63 (12)
C4—C5—C6	121.20 (14)	C14—C13—C12	119.65 (18)
C4—C5—H5	119.4	C14—C13—H13	120.2
C6—C5—H5	119.4	C12—C13—H13	120.2
C5—C6—C1	118.97 (13)	C13—C14—C15	120.53 (17)
C5—C6—C7	120.44 (13)	C13—C14—H14	119.7
C1—C6—C7	120.58 (12)	C15—C14—H14	119.7
O1—C7—C6	124.58 (15)	C16—C15—C14	120.0 (2)
O1—C7—H7	117.7	C16—C15—H15	120.0
C6—C7—H7	117.7	C14—C15—H15	120.0
O2—C8—C9	107.51 (11)	C15—C16—C11	121.09 (18)
O2—C8—H8A	110.2	C15—C16—H16	119.5
C9—C8—H8A	110.2	C11—C16—H16	119.5
O2—C8—H8B	110.2	N1—C17—C9	178.10 (17)
C9—C8—H8B	110.2		
			
C8—O2—C1—C2	−21.16 (19)	C17—C9—C10—C11	−177.54 (15)
C8—O2—C1—C6	160.05 (12)	C8—C9—C10—C11	0.5 (3)
O2—C1—C2—C3	−176.06 (14)	C9—C10—C11—C16	−37.5 (2)
C6—C1—C2—C3	2.7 (2)	C9—C10—C11—C12	143.27 (17)
C1—C2—C3—C4	−0.3 (3)	C16—C11—C12—C13	1.2 (2)
C2—C3—C4—C5	−1.7 (3)	C10—C11—C12—C13	−179.48 (15)
C3—C4—C5—C6	1.4 (3)	C16—C11—C12—Cl1	179.72 (12)
C4—C5—C6—C1	0.9 (2)	C10—C11—C12—Cl1	−1.0 (2)
C4—C5—C6—C7	−177.70 (15)	C11—C12—C13—C14	−0.4 (3)
O2—C1—C6—C5	175.89 (12)	Cl1—C12—C13—C14	−178.89 (14)
C2—C1—C6—C5	−2.9 (2)	C12—C13—C14—C15	−0.3 (3)
O2—C1—C6—C7	−5.54 (19)	C13—C14—C15—C16	0.2 (3)
C2—C1—C6—C7	175.62 (13)	C14—C15—C16—C11	0.7 (3)
C5—C6—C7—O1	0.0 (3)	C12—C11—C16—C15	−1.4 (2)
C1—C6—C7—O1	−178.53 (16)	C10—C11—C16—C15	179.34 (15)
C1—O2—C8—C9	−173.38 (11)	C10—C9—C17—N1	36 (6)
O2—C8—C9—C10	−149.36 (14)	C8—C9—C17—N1	−142 (6)
O2—C8—C9—C17	28.70 (17)		
